# Bilateral hypotropic dissociated vertical deviation: a case report 

**DOI:** 10.1186/s13256-020-02567-7

**Published:** 2020-12-10

**Authors:** Nawar Shohadeh, Nawras Alhalabi

**Affiliations:** 1 Specialized Center for Strabismus, Damascus, Syria; 2 Al Zahraa Center for Perfect Vision, Damascus, Syria; 3 Department of Ophthalmology, Faculty of Medicine, Damascus University, Damascus, Syria; 4 Department of Internal Medicine, Faculty of Medicine, Syrian Private University, Damascus, Syria

**Keywords:** Strabismus, Hypotropic, Dissociated vertical deviation, Nystagmus, Case report

## Abstract

**Background:**

Hypotropic dissociated vertical deviation (DVD) is a special form of strabismus characterized by a slow drift of the non-fixating eye when the other eye is fixating on a target. In contrast to hypertropic DVD, which is common, hypotropic DVD is exceedingly rare and seldom reported in previous literature. In this case, we report the clinical features of a rare case of a Syrian child with bilateral hypotropic DVD accompanied by manifest latent nystagmus and intermittent exotropia.

**Case presentation:**

A 4-year-old Syrian Arab girl presented with intermittent exotropia of both eyes since the age of 7 months, without any prior treatment. The fixation was alternating. She had manifest latent nystagmus in both eyes and anomalous head posture. She had bilateral hypotropic DVD in both eyes which only appeared when covering each eye. The patient underwent bilateral lateral rectus recession with posterior fixation and bilateral inferior oblique recession. Three months after surgery, she was orthophoric in the primary gaze position with a normal head posture. No alteration of the appearance of the hypotropic DVD was observed after the surgery.

**Conclusion:**

This is a rare case of hypotropic DVD showing bilateral hypotropic DVD with different characteristics from those previously reported cases (bilateral hypotropic DVD with intermittent exotropia, dissociated horizontal deviation, manifest latent nystagmus, and bilateral inferior oblique overaction). The hypotropic DVD only appeared when covering each eye, and thus there was no need for surgery. Moreover, the inferior oblique recession did not seem to negatively affect the appearance of the eyes.

## Background

Dissociated vertical deviation (DVD) is a unique form of strabismus characterized by slow vertical drift of the non-fixating eye when the other eye is fixating on a target or when covering the eye. Hypertropic DVD is common and usually accompanies congenital esotropia and latent nystagmus [1–3]. Hypotropic DVD is a very rare condition and has also been called dissociated hypotropia, inverse DVD, or fixation-linked hypotropia [3]. Only ten cases have been reported in the literature [4], in addition to four known unpublished cases [2]. To the best of our knowledge, this is the first reported case in Syria.

Most previous cases reported hypotropic DVD as frequently being unilateral with an acquired etiology. Reported causes were intraocular injuries, high myopia, anisometropia, and poor best-corrected visual acuity (BCVA) [3]. The mechanism of hypotropic DVD is generally unknown, with many hypotheses postulated [3, 5, 6].

In this case, we report a Syrian child with bilateral hypotropic DVD accompanied by manifest latent nystagmus and intermittent exotropia.

## Case presentation

A 4-year-old Syrian Arab girl presented with intermittent exotropia of both eyes since the age of 7 months, without any prior treatment. She appeared to be in good health.

Cyclorefraction was +1.00 +1.00 × 85° for her right eye, and +1.00 +1.00 × 65° for the left eye. The girl was not cooperative for visual acuity testing. The fixation was alternating. The exodeviation was very variable due to significant dissociated horizontal deviation (DHD) in both eyes.

There was bilateral inferior oblique overaction (+2) along with bilateral superior oblique underaction (−1) and significant V-pattern. She had manifest latent nystagmus in both eyes that significantly decreased in the downgaze.

**Fig. 1:**
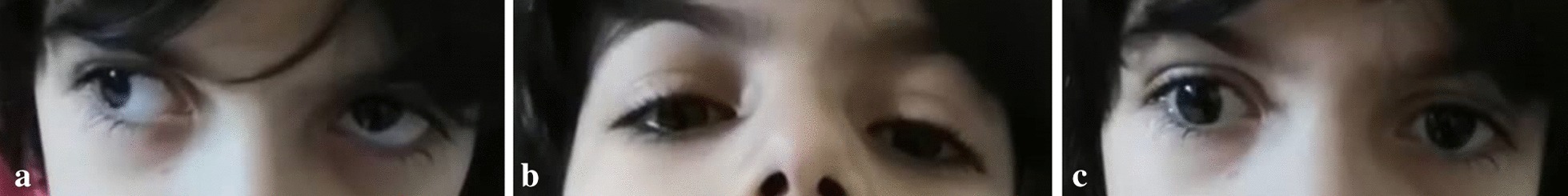
Patient's strabismus in some positions of gaze. **a** Upgaze—exotropia 75 prism diopters; **b** Downgaze—intermittent exotropia 5 prism diopters; **c** Primary position—intermittent exotropia 30 prism diopters

She had an anomalous head posture: chin up (downgaze) to inhibit nystagmus and exotropia. Bilateral hypotropic DVD in both eyes only appeared when covering each eye (see Additional file 1). The patient's strabismus in some positions of gaze is shown in Fig. 1. Exodeviation measurements in different positions of gaze are summarized in Table 1.

**Table 1:** Exodeviation measurements in different positions of gaze

Primary position measurements					
		Near		Far	
Horizontal		−30		≈ −50	
Vertical		0		0	

Near deviation in different positions of gaze					
Horizontal tropia			Vertical tropia		
	−75			0	
−30	−30	−30	−18	0	+25
	−5			0	
			+8 R tilt		−5 L tilt

The patient underwent bilateral lateral rectus recession 4 mm with posterior fixation technique (lateral rectus recession of 11 mm with 7 mm resection) and bilateral inferior oblique recession of 10 mm.

Three months after surgery, she was orthophoric in the primary gaze position with a very mild V-pattern and very mild residual right inferior oblique overaction. The patient also had a normal head posture. No alteration in the appearance of the hypotropic DVD was observed for either eye after the surgery, and it still appeared only by covering each eye (see Additional file 2).

## Discussion and conclusion

Hypotropic DVD is characterized by a dissociated hypo-deviation of one eye that presents intermittently only while the other eye is fixating on a target.

Kraft *et al*. were the first to describe a case in 1999, and they reported two cases; the first was associated with high myopic anisometropia, the second with a penetrating ocular injury [2]. Kowal and Kumar described two other cases that were associated with high myopic anisometropia [7]. Greenberg and Pollard reported an additional case as a result of an infectious neurologic brain injury, which is the only published bilateral case [1]. Lim reported four cases; two had high myopic anisometropia, and the other two were associated with penetrating ocular injury [3]. Rajavi *et al*. reported the last published case in 2013, a female with unilateral esotropia, hypotropic DVD, and mild amblyopia [4]. A summary of all published cases is shown in Table 2.

**Table 2:** Summary and characteristics of previously published cases

Paper	Case	Age (years)	Gender	BCVA	Laterality	Deviation amount (PD)	Surgery
Kraft *et al.* 2000 [8]	1	14	M	R (HM), L 6/6	Unilateral	20	IR recession
Greenberg and Pollard 2001 [1]	2	2	M	Not measured	Bilateral	Not measured	
Kowal and Kumar 2003 [7]	3	52	F	R 6/4, L 6/16	Unilateral	25	
	4	35	F	R 6/8, L 6/60	Unilateral	30	IR combined recession–resection
Kraft *et al.* 2006 [2]	5	13	M	R 6/6, L 6/60	Unilateral	18	IR recession
Lim 2008 [3]	6	40	M	R 6/120, L 6/6	Unilateral	30	IR recession
	7	25	F	R 6/240, L 6/6	Unilateral	25	IR combined recession–resection
	8	27	M	R 6/240, L 6/6	Unilateral	25	
	9	27	M	R 6/6, L 6/7.5	Unilateral	30	IR combined recession–resection
Rajavi *et al.* 2013 [4]	10	25	F	R 6/7.5, L 6/12	Unilateral	40	MR recession with superior tendon transposition
Shohadeh and Alhalabi 2020 (this case)	11	4	F	Not measured	Bilateral	30	LR recession with posterior fixation, IO recession

*BCVA* best-corrected visual acuity, *F* female, *HM* hand movement, *IO* inferior rectus, *IR* inferior rectus, *LR* lateral rectus, *M* male, *MR* medial rectus, *PD* prism diopter

All reported cases except one were unilateral. The only previously published bilateral case was related to an acquired neurologic injury, in contrast to the other unilateral cases. Two additional unpublished bilateral cases were previously mentioned in the literature [2]. Our case is also bilateral.

The most prominent feature in all cases of unilateral hypotropic DVD was a vision deficit in the hypo-deviated eye. All but two cases [3, 4] had some level of severely impaired vision. The causes of low visual acuity included deep amblyopia associated with high myopic anisometropia or penetrating ocular trauma.

Generally, a certain amount of difference in visual acuity between the two eyes commonly exists in cases of hypotropic DVD, although the degree of difference may be quite variable [4]. Our patient was not cooperative for visual acuity testing; however, the alternating fixation implies the presence of comparable levels of visual acuity in both eyes.

Hypotropic DVD seems to have an acquired etiology in all previous cases; therefore, they did not have latent nystagmus. The exception is the case reported by Rajavi *et al*., which had latent nystagmus, and the authors mentioned that the etiology was controversial [3, 4]. Our case appears to have a congenital etiology accompanied by manifest latent nystagmus, which is characteristic of hypertropic DVD.

Hypotropic DVD is similar to hypertropic DVD in various aspects of oculomotor characteristics. Similarities between these two conditions include slow vertical drift and returning to the primary gaze position of either eye, the dissociated nature of the deviation depending on the fixation status, intermittence of the deviation depending on the fusional status, and possible association with horizontal strabismus [3]. In our case, we noticed other similar aspects such as the association with latent nystagmus, bilateralism, and perhaps congenital etiology.

In conclusion, this is a rare case of hypotropic DVD showing bilateral hypotropic DVD with intermittent exotropia, dissociated horizontal deviation, manifest latent nystagmus, and bilateral inferior oblique overaction. The hypotropic DVD only appears when covering each eye; thus, there is no need for surgery. Moreover, the inferior oblique recession does not seem to negatively affect the appearance of the eyes.

## Supplementary information

**Additional file 1.** Strabismus examination at the presentation.

**Additional file 2.** Strabismus examination after the surgery.

## Data Availability

Not applicable.

## Abbreviations

BCVA: Best-corrected visual acuity
DVD: Dissociated vertical deviation
